# Operationalizing recipient-centeredness: three facets of values-driven care in syringe services programs

**DOI:** 10.1186/s43058-026-01051-5

**Published:** 2026-07-28

**Authors:** Elana Forman, William H. Eger, Christopher F. Akiba, Sheila V. Patel, Shelby L. Huffaker, Jessica Smith, Barrot H. Lambdin, Alexis M. Roth, Angela R. Bazzi

**Affiliations:** 1 Dornsife School of Public Health, Drexel University, Philadelphia, PA USA; 2 School of Social Work, San Diego State University, San Diego, California, USA; 3 School of Medicine, University of California San Diego, La Jolla, California, USA; 4 RTI International institution, Research Triangle Park, NC USA; 5 Herbert Wertheim School of Public Health, University of California San Diego, 9500 Gilman Drive, La Jolla, CA 92093 USA; 6 Boston University School of Public Health, Boston, MA USA

**Keywords:** Substance use, Harm reduction, Consolidated framework for implementation research, Recipient-centeredness, Low-barrier care, Organizational culture, Trust, Health services

## Abstract

**Background:**

Syringe services programs (SSPs) offer critical, evidence-based services for people who use drugs (PWUD), including infectious disease and overdose prevention. SSPs operate from harm reduction and health equity frameworks, prioritizing non-coercive care and client autonomy; these values foster engagement among a community often stigmatized or discriminated against in traditional healthcare settings. As SSPs increasingly expand their clinical service offerings, they may encounter tensions among their core values, internal or funder-driven pressures for service expansion, and partnerships with external healthcare systems. Accordingly, we undertook a qualitative study to understand how SSP staff uphold core values and practice recipient-centeredness, as defined by the Consolidated Framework for Implementation Research (CFIR), when engaging PWUD in health services within harm reduction settings.

**Methods:**

From May 2023 to February 2024, we conducted qualitative interviews with 41 representatives of 27 SSPs serving diverse U.S. jurisdictions. Guided by the CFIR, trained interviewers used semi-structured interview guides to explore SSPs’ health services delivery models. From interviews, recipient-centeredness emerged as a central theme, leading us to employ thematic and narrative analysis to further explore how SSPs defined and operationalized this construct in their programs’ health-related services.

**Results:**

SSP staff consistently emphasized the centrality of core values guiding their services, particularly recipient-centeredness. SSPs operationalized recipient-centeredness through three primary facets: accessibility, consistency, and non-coerciveness. These facets informed direct service delivery and referral processes, enabling programs to build trust and meet clients’ complex needs. To uphold these facets, SSPs implemented low-barrier service access, mobile outreach, client-centered referrals, and predictable service schedules despite dynamic and sometimes unsupportive contexts.

**Conclusions:**

This study offers a specific operationalization of recipient-centeredness for the unique and understudied setting of SSPs, identifying accessibility, consistency, and non-coerciveness as key facets of this organizational culture. Understanding these facets yields actionable guidance for supporting SSPs in implementing values-driven public health and prevention programming. These findings may also be relevant for other harm reduction and low-barrier care settings seeking to preserve trust, dignity, autonomy, and effectiveness while delivering evidence-based services.

**Trial registration:**

NCT06025435

Contributions to the literature

- This study applies the Consolidated Framework for Implementation Research (CFIR) to empirically characterize how recipient-centeredness is understood and operationalized within syringe services programs (SSPs), a community-based service delivery setting essential for reducing infectious disease transmission and overdose mortality from substance use.
- Qualitative interviews and analysis revealed three facets of recipient-centeredness that inform SSPs’ engagement: accessibility, consistency, and non-coerciveness
- Findings highlight the need to support SSPs and, potentially, other low-barrier care systems in implementing values-driven public health and prevention programming that maintains recipient-centeredness.

## Background

People who use drugs (PWUD) experience a range of health-related harms, including overdose, infectious diseases, and complex wounds [1–4]. However, this population often encounters stigma, discrimination, and other structural barriers that limit access to traditional healthcare settings, including primary care [5–7], and contribute to delayed treatment and reduced uptake of evidence-based preventative services [8].

Due to barriers PWUD face in traditional healthcare settings, syringe services programs (SSPs) have become a cornerstone of public health efforts to address the health needs of this community by offering a de-stigmatizing approach to service provision [9]. Grounded in principles of harm reduction and health equity, many SSPs are also deeply engaged in social justice efforts aimed at advocating for the rights and dignity of PWUD [10]. Most SSPs offer a wide array of evidence-based services, including overdose education and naloxone distribution, safer drug use supply distribution, infectious disease testing, and substance use treatment referrals [11]. Some SSPs directly offer more clinically advanced health services like human immunodeficiency virus (HIV) and hepatitis C virus (HCV) treatment, wound care, mental health care, and medications for opioid use disorder [11–15]. Over time, more SSPs have expanded their scope to include these clinical health services, either by integrating clinicians into their staff or partnering with external providers to offer co-located services [16–18]. However, because of the numerous challenges to expanding services, including funding and staff capacity limitations, many SSPs still rely on referrals to external providers for specialized services [11, 19, 20].

Given the success of SSPs in reaching vulnerable groups of PWUD and engaging them in prevention, care, and treatment, there is growing interest in supporting SSPs to expand the health services they offer [21, 22]. However, increasing the clinical scope and medicalization of SSPs may conflict with their core principles, including those related to centering clients in care delivery [22–25]. For example, as SSP staff attempt to integrate more specialized health services (or increasingly refer their clients to such services), they may confront distal systems and providers that do not share the same values. This tension, combined with the pervasive stigmatization PWUD experience in traditional healthcare settings, underscores the need for research on how SSP staff approach health services delivery for their clients, including the integration of new onsite health services or referrals to external healthcare providers.

Because of these tensions and other complexities of SSPs as a service delivery setting, implementation frameworks such as the Consolidated Framework for Implementation Research (CFIR) have increasingly been used to examine factors influencing implementation of service delivery within SSPs [26–29]. CFIR includes 48 constructs across five domains: innovation, outer setting, inner setting, individuals, and implementation processes [30]. Despite popular use of CFIR to categorize implementation determinants, the framework remains vague in some of its definitions and operationalizations [31]. In fact, a common challenge in applying CFIR is ambiguity around construct definitions [31, 32].

Among CFIR constructs particularly relevant to the SSP setting is recipient-centeredness, which aligns with SSPs’ emphasis on “meeting people where they are,” or working with clients in ways that respect and respond to the priorities, autonomy, and self-defined goals [30]. Despite the centrality of recipient-centeredness to harm reduction practice, to our knowledge, no research exists that has examined how SSP staff define and operationalize this construct in the context of delivering or expanding health services. Thus, while CFIR has been useful for categorizing multilevel implementation determinants, a lack of descriptive clarity challenges the utility of some of its components.

A more thorough understanding of recipient-centeredness may enhance the utility of CFIR for examining a cornerstone of harm reduction service provision. Thus, we conducted a qualitative analysis of interview data collected from an ongoing implementation trial focused on HIV services delivery within U.S. SSPs (ClinicalTrials.gov: NCT06025435). Our goal was to understand how SSP staff defined and operationalized recipient-centeredness in the context of delivering health services. Because harm reduction values—including autonomy, dignity, nonjudgment, and low-barrier access—are central to SSPs’ practice, clarifying how staff operationalize recipient-centeredness may inform efforts to support health service delivery without compromising these core principles.

## Methods

### Study design & sample

From May 2023 to February 2024, we conducted qualitative interviews with 41 representatives from 27 U.S. SSPs as part of a parent study focused on understanding programs’ HIV services implementation [26]. We used purposive sampling [33] to identify SSPs across four geographic regions delineated in CDC’s National HIV Surveillance Report (West, South, Midwest, Northeast) [34] with diversity in program type (e.g., community-based program, health department). We drew on our professional networks and the public directory of the North American Syringe Exchange Network [35] to contact program directors by email to recruit staff familiar with their programs’ HIV-related services delivery. Because many programs had multiple staff interested in participating who were knowledgeable about HIV services, we arranged individual or small group interviews (i.e., up to three staff could participate in a single interview representing one program). Interview participants provided verbal informed consent; SSPs received $200 compensation for staff participation. The institutional review boards of the University of California San Diego and Drexel University reviewed and approved the study protocol and provided a waiver of documentation of informed consent. This manuscript was prepared with reference to the Standards for Reporting Qualitative Research.

### Data collection

We emailed interviewees a link to a brief online survey to capture basic program characteristics; interviewers reviewed survey responses prior to qualitative interviews for general background and contextual information (Table 1). Next, we conducted qualitative interviews via Zoom video conferencing using a semi-structured interview guide informed by existing literature and CFIR [30]. CFIR informed questions related to inner- and outer-setting contexts and how they affected delivery of different health services, while existing literature informed probes about barriers, constraints, and facilitators faced by programs. We pilot-tested the guide in one interview and discussed revisions with all team members. Subsequent interviews were conducted by two trained, graduate-level members of our research team: one who primarily served as the interviewer and one who took detailed notes (described below). Interviews lasted approximately one hour and were digitally recorded for professional transcription. We verified transcripts for accuracy and de-identification following a structured protocol.

**Table 1:** Participant and Program Characteristics

1A. Participant Characteristics	
**Participant role/position** (*n* = 41*)	
Leadership (Director, Manager, Coordinator, Administrator)	26
Frontline staff (Outreach, Public Health Worker)	9
Clinical staff (Nurse, Phlebotomist, Tester, Medical Director)	4
Case management (Care Coordinator, Peer Counselor)	2
**1B. Program Characteristics** (*n* = 27)	
**Geographic Region**	
West	7 (25.9)
Midwest	5 (18.5)
South	8 (29.6)
Northeast	7 (25.9)
**Located in EHE jurisdiction**	15 (55.6)
**Number of staff**	
≤10	8 (29.6)
11–30	9 (33.3)
>30	10 (37.0)
**Number of unduplicated clients per month**	
≤100	3 (12.0)
101 - 400	10 (40.0)
401–1000	9 (36.0)
>1000	3 (12.0)
Unsure/Not Reported	2 (7.4)
**Service delivery modalities**	
Fixed services only	2 (7.4)
Mobile services only	4 (14.8)
Both fixed and mobile services	21 (77.8)
**SSP classification**	
Community-based program	17 (63.0)
Municipal health department	7 (25.9)
Academic or healthcare system	3 (11.1)
**Direct services provided**	
Naloxone distribution	27 (100.0)
Sterile syringes	27 (100.0)
Safer smoking supplies	19 (70.4)
Safer sex supplies	27 (100.0)
HIV testing	21 (77.8)
PrEP or PEP	10 (37.0)
HIV treatment	7 (25.9)
Hepatitis, STI, or other testing	20 (74.1)
Hepatitis, STI, or other treatment	9 (33.3)
Basic clinical services	13 (48.1)
Mental health services	8 (29.6)
Case management and/or housing coordination	14 (51.9)
Other health services not specified on this list**	9 (33.3)
**Referrals provided** (*n* = 25***)	
HIV testing	13 (48.1)
PrEP or PEP	21 (77.8)
HIV treatment	20 (74.1)
Hepatitis, STI, or other infectious disease testing	13 (48.1)
Hepatitis, STI, or other infectious disease treatment	20 (74.1)
Basic clinical services	19 (70.4)
Mental health services	22 (81.5)
Case management and/or housing coordination	16 (59.3)
EHE, Ending the HIV Epidemic; PEP, post-exposure prophylaxis; PrEP, pre-exposure prophylaxis; SSP, syringe services program; STI, sexually transmitted infection *The number of participants is greater than number of programs because multiple people could partake in each interview **MOUD provision or referral was not assessed as a discrete survey item and may be included under “other health services” ***Two programs skipped or declined to answer this question	

### Data analysis

During and immediately following interviews, interviewers and note takers completed detailed notes using a structured debriefing report with fields for key and emerging topics [36]. Our analytic team included researchers with graduate-level training in implementation science and public health. All team members had prior experience collaborating with SSPs, either in research or practice (including direct service delivery), which facilitated sensitivity to participants’ perspectives but also required reflexive attention to our assumptions about values-driven care. Throughout the analysis, we engaged in memo writing and team discussions to reflect on how our positionalities informed theme development.

Although participants were not directly asked about recipient-centeredness as a CFIR construct, in the first stage of the analytic process, we noticed participants repeatedly described core harm reduction values (e.g., low-barrier access, autonomy, nonjudgment, trust, and “meeting people where they are”) as shaping health services delivery. We interpreted these accounts through CFIR’s recipient-centeredness construct, which concerns shared values, beliefs, and norms around caring for and addressing recipients’ needs [30]. Using a reflexive thematic analysis approach [37] grounded in team discussions and debriefing reports, we created a codebook with eight in vivo codes capturing values and strategies staff described (e.g., codes for “meeting people where they are,” “low-barrier access,” “building trust,” “autonomy/nonjudgment”). We then compared these coded excerpts across programs to identify broader patterns in how SSPs operationalized recipient-centeredness. Because the present manuscript focuses on this emergent construct, CFIR-informed inner- and outer-setting codes from the parent analysis [30] helped contextualize program narratives and are not presented as formal results. After reaching consensus on a final codebook, two analysts independently applied codes to all transcripts using Dedoose [38], meeting regularly to compare code application for consistency. After double-coding all transcripts, we organized excerpts into a matrix to observe within- and between-case themes and distinctions. Through analysis of the matrix, we extracted key themes related to the values, strategies, and delivery practices through which programs operationalized recipient-centeredness.

Additionally, we reviewed transcripts, debriefing reports, and public-facing mission statements of SSPs to compose case study narratives [39] for all 27 programs. These narratives were developed by synthesizing and triangulating data about programs to summarize priorities, service delivery approach, and operational contexts. We identified key facets of recipient-centeredness by looking at both the matrix of coded data and program-specific narratives. From this integration of thematic and narrative analysis, we found patterns related to the themes described below.

## Results

### Sample Characteristics

We interviewed 41 staff from 27 SSPs that ranged in geographic region, program type (e.g., health department, nonprofit), and size (Table 1). All programs provided syringes, naloxone, and safer sex supplies. Direct provision of health and social services by the SSPs or on-site partners varied, with 21 (78%) providing HIV testing, 20 (74%) providing HCV, sexually transmitted infections (STIs), or other infectious disease testing, 14 (52%) providing case management or housing coordination services, 13 (48%) providing basic clinical care, and 8 (30%) providing mental health care. Referrals to external health services also varied, with 13 (48%) referring out for infectious disease testing (i.e., for HIV, HCV, STIs, or other infections), 20 (74%) referring to infectious disease treatment, 21 (78%) referring to pre- or post-exposure prophylaxis for HIV prevention, 19 (70%) referring to basic clinical services, and 22 (82%) referring to mental health services.

### Facets of recipient-centeredness

Although programs were asked broadly about health services delivery, recipient-centeredness [30] emerged as a critical component of how SSP staff described their work. Staff emphasized three distinct facets of recipient-centeredness (1): accessibility (2), consistency, and (3) non-coerciveness. Programs relied on these facets to prioritize recipient-centeredness during health services delivery in a complex and dynamic service environment.

#### Accessibility

Regardless of which health services were provided, programs prioritized improving clients’ ability to access them (accessibility). Conceptualizations of accessibility were broad, extending beyond the physical to encompass social, financial, and emotional barriers to service engagement. As one staff member explained:

*We strive for all of our programs to be incredibly low barrier and wrap around [for] folks with complex needs … cognitive issues, physical health issues, mental health, active substance use. [We] just really strive to meet people exactly where they’re at in the most low-barrier way, honoring people’s choices and decisions.* (Program 10).

Operationalizing accessibility often included mobile outreach, which nearly all programs provided, “*because a lot of people we see on outreach don’t make it to the building.*” (Program 1). In fact, many tailored their outreach efforts to reach additional individuals who could benefit from their services: “*If we hear on outreach that there’s a lot of people in a different area and it’s relatively close to where we usually go, we’ll just drive over there in the van and check it out*.” (Program 1).

Programs ranged in how many services they provided directly compared to offering referrals, with several providing few to no clinical services themselves. Even when external referrals were the primary mechanism for linking clients to specialty health services, making those referrals accessible was a core strategy for practicing recipient-centeredness: *“Some [clients] need you to literally go to the appointments with them; others can navigate the system themselves. That’s [where] the whole ‘meeting the participant where they’re at’ kind of thing comes into play.”* (Program 16). Overall, programs sought to “*try to make [those] referral[s] as easy as possible*.” (Program 23).

Accessibility also extended to the interpersonal level, where approachability of staff and degree of comfort felt by clients played a crucial role in offering health services, whether directly or via referrals:

*People’s situations are unique and it can be hard to build relationships and trust. But if you can do that well, usually with incentives, [like gift] cards, and just being nice, having conversations with people, just saying “hi,” [it] can open up a conversation. Once you start talking, you’ll feel when you can get into that [health issue] with that person, you know, you’ll feel the trust [and] they’ll let you know”.* (Program 1).

These efforts to reduce barriers for clients not only facilitated physical access, but also fostered the rapport and trust needed to promote engagement, crucial elements for programs’ practice of recipient-centeredness.

#### Consistency

Across programs, delivering services consistently (consistency) was considered key to fostering trust and increasing client engagement in health services. Consistency was conceptualized as having core services available (e.g., syringe services, overdose education and naloxone distribution), even when few or no clients were present: *“One thing about being mobile is whether you’re busy or not, it’s very important to stay consistent, to go out every day with that mobile unit so they can see that you’re always there if they ever need you.”* (Program 9). Staff recognized that predictability and stability were key factors in helping gain community trust over time, with some noting that “*it took about a year for our services to really be trusted by people who use drugs in our county, and even then, there are subsets of the population that are distrustful …”* (Program 5).

Consistency also meant having services available when people were ready for them: “*People are passing through; they hear about us and they stop by when they’re traveling somewhere. So, if we don’t have what they need right there in that minute, we lose them*.” (Program 25). Some staff expanded on this idea by offering “*more nontraditional hours in different areas*” (Program 18) or having on-call medical providers that could “*come in outside of [regular clinical] hours.*” (Program 25). For these programs, being consistently available was a cornerstone of recipient-centeredness. Interviewees explained that, as their programs sought to expand their health services, prioritizing consistency was often more important than offering new services: “*So we’ve seen our program grow [and] expand, but what helps us the most is that we are consistent, and we are there*.” (Program 21).

For some programs, this meant intentionally *not* expanding services and instead prioritizing consistency with existing services. These programs would often partner with other organizations with trustworthy providers who could help make services available to their clients while SSPs focused on services they felt equipped to do: “*There’s just so much mistrust in the healthcare system that a lot of it is starting to come down to us being able to streamline services to providers that we know*” (Program 16) and “*working with our partner organizations, there are more or less things that we can do.”* (Program 21).

For programs relying on referrals, consistency also meant reliably supporting, following up on, and tailoring referrals to clients’ needs:

*If the individual is engaged with us in any capacity, then [our] case management will likely follow up with them [and say], “Hey, I know you had an HIV appointment, were you able to make that? Oh, you need a ride? I can get you a ride.” So, we can definitely support them getting care elsewhere.* (Program 20).

Consistency helped programs ensure continuity of care by providing clients with a trusted, stable place to access services and referrals. Thus, having core services consistently available and maintaining a stable presence in the community were key facets of programs’ recipient-centeredness.

#### Non-Coerciveness

The third facet of recipient-centeredness involved creating an environment that respected clients’ autonomy, offering health services in a manner that did not impose judgment or pressure, and allowing clients to engage in services at their own pace (non-coerciveness): “*You know, our Modus Operandi is ‘let the person know what we have,’ which is why we have all the signage and stuff, then let them direct the engagement.”* (Program 4).

Non-coerciveness was particularly important for programs working with individuals who may have experienced discrimination, trauma, or stipulations around ceasing substance use while seeking care in traditional healthcare settings. In contrast, SSP staff tended to focus on building trusting relationships where clients could make informed decisions about their care without fear of coercion:

*We just try to tell them, “Hey, you don’t have to do A, B, and C,” but, again, they’re all adults, and we can’t force them to do stuff when they’re not ready. But they do come to us when they are ready and they’ve had enough and they’re tired and they want to go to detox, then we’re more than welcome to usher them to where they need to get into … you have to meet the client where they are.* (Program 18).

*I think there’s a lot of very honest, caring conversations happening with people around what they’re doing, and the stage is set for a non-judgmental space to talk about sex work or other sort of high risks. Then folks are given education, explanation, and encouragement.* (Program 10).

Such encouragement was deployed without “*push[ing] people because we know pushing pushes them away*.” (Program 4).

Beyond health services and referrals, non-coerciveness extended to the ways that programs prioritized clients’ autonomy:

*I think for us a big thing is autonomy, making sure we’re really letting people make informed decisions about their own lives, especially given the folks we serve. It’s really helping no one to set a level of morality [or expectations] around what people are or how people are using. [illicit substances]* (Program 27).

For programs primarily offering referrals and fewer direct services, staff vetted referral networks to ensure they were sending clients to places that similarly prioritized these values:

*We basically have a very specific list [of] where [and] who we’re sending you to. The reason that we send them to [those places] is because [their providers] know and have agreed that “I’m not going to be spending all of my time trying to convince someone you send me that they have to stop using for me to treat them”.* (Program 22).

Voluntary models of care respected clients’ self-determination and readiness to engage with services ranging from infectious disease testing and preventative services to treatment for infections, substance use disorders, or other mental health conditions. Programs’ commitment to non-coercion as a core facet of recipient-centeredness allowed for a safer, more supportive care space where clients could build trust and, over time, engage with desired services meaningfully.

Together, these three facets provide a more granular understanding of how recipient-centeredness is operationalized within SSPs, illustrating how harm reduction values can be translated into everyday implementation practices.

## Discussion

Our study contributes to a growing body of research examining how SSPs deliver health services, with a specific focus on the values that guide staff-client engagement, mitigate stigma, overcome structural fragmentation, and foster trust among a community that is routinely marginalized in mainstream care [8, 40]. Although our interview guide was not originally designed to explore “recipient-centeredness” in depth, the concept emerged as a central theme across interviews, which we subsequently interpreted using CFIR [32].

While CFIR conceptualizes recipient-centeredness as part of organizational culture, it remains relatively vague in describing how values are enacted in practice [31, 41]. One of the primary challenges in using CFIR is the ambiguity of some constructs [32]. Our findings contribute to the applied use of this construct in SSPs by empirically identifying three operational facets—accessibility, consistency, and non-coerciveness—through which SSPs actualize recipient-centeredness in everyday service delivery. These facets extend beyond broad principles to offer concrete practices: accessibility encompassed physical, emotional, and logistical dimensions enacted through mobile outreach, rapport-building, and warm handoffs; consistency fostered sustained trust through predictable presence and reliable follow-up; and non-coerciveness ensured clients engaged on their own terms, honoring autonomy and readiness. By articulating these dimensions, we provide an SSP-specific operationalization of recipient-centeredness that may help implementation researchers and practitioners apply this CFIR construct more concretely in harm reduction and other low-barrier service settings.

Building on this operationalization of recipient-centeredness, it is important to consider how these findings can inform implementation strategies and advance the broader implementation science field. Our findings resonate with broader harm reduction and trauma-informed care frameworks [23], including the U.S. Substance Abuse and Mental Health Services’ (SAMHSA) harm reduction framework which highlights strategies and practices to center clients [42], and contribute to a growing conversation in implementation science around how care grounded in explicit organizational values can be theorized and operationalized [43, 44]. In these contexts, our findings offer concrete, actionable pathways for designing delivery models that uphold recipient-centeredness, such as changing service sites to increase access, shifting or revising professional roles among those offering care, or involving clients in implementation efforts [45]. For SSPs, operationalization of such delivery systems can include mobile outreach, peer-led navigation and “tele-harm reduction” [46–48]. These implementation strategies may be particularly important in SSPs and other low-barrier settings and may also have relevance for systems serving communities frequently excluded or stigmatized in healthcare, including people living with HIV, lesbian, gay, bisexual, transgender or queer (LGBTQ+) individuals, sex workers, and others [49–51].

Implementation researchers and health system leaders can build on our findings by further exploring these facets of recipient-centeredness and how they influence implementation and delivery of services in additional contexts. Doing so could further clarify how recipient-centeredness functions in harm reduction and other settings serving communities disproportionately affected by structural vulnerabilities. These operational descriptions can also inform pre-implementation assessments, guide partnership-building across clinical and community-based settings, and help define what it means to deliver care that is recipient-centered.

Finally, our findings should still be interpreted within the broader structural context in which SSPs operate. Prior literature documents that SSPs often face substantial internal and external challenges, including political hostility, local restrictions, stigmatizing public attitudes, staffing shortages or burnout, limited infrastructure, and competing demands [52–55]. These barriers are not necessarily unique to SSPs, but they are particularly consequential in settings where relational trust is a primary driver of engagement; they are also best understood not as programmatic deficiencies, but as symptoms of chronic underinvestment in harm reduction infrastructure [53, 56]. These constraints mirror longstanding philosophical conflicts between harm reduction and traditional medical models [57], as well as broader implementation science work highlighting the importance of political will, community buy-in, and supportive conditions for sustaining evidence-based practices [58–60]. Without supportive policy frameworks, stable funding, and cultural alignment across service systems, even committed programs may face challenges delivering services in ways that fully align with harm reduction values. Future research should directly examine how inner- and outer-setting barriers shape SSPs’ ability to sustain accessibility, consistency, and non-coerciveness while expanding HIV and other health-related services.

Our study is not without limitations. First, our sample was not designed to be representative and only included SSPs participating in a pre-implementation study for an HIV-focused implementation trial, which may mean they are more clinically aligned or resourced than other programs. Second, we interviewed program staff and not clients, whose perspectives would provide critical insights into whether services are experienced as accessible, consistent, and non-coercive. Additionally, our original study guide was not designed to explore recipient-centeredness, and instead we explored an emergent theme from our larger parent study. Finally, social desirability bias may have influenced how interviewees described their engagement strategies or challenges. Nonetheless, our use of in-depth narrative data across a diverse national sample allowed us to identify meaningful patterns in how recipient-centeredness is understood, practiced, and operationalized within SSPs. Future research can build on our work by more directly examining barriers SSPs face in sustaining accessibility, consistency, and non-coerciveness, given the importance of these facets in this delivery setting, and defining and operationalizing recipient-centeredness and other CFIR constructs in other, varied settings.

## Conclusions

SSPs operate at the intersection of public health and social justice, expanding health service provision and linkages while navigating significant structural constraints. Our findings identify three concrete facets of recipient-centeredness—accessibility, consistency, and non-coerciveness—through which SSPs translate core values into everyday practice. Rather than offering a universal definition of recipient-centeredness, these empirically derived facets provide practical guidance for SSPs and other low-barrier, harm reduction-oriented settings seeking to promote trust, autonomy, and engagement among people who use drugs. Supporting a culture of recipient-centeredness is therefore essential for those seeking to partner with, fund, or regulate SSPs, and for implementation science efforts aimed at more equitable and sustainable service delivery.

## Data Availability

Due to ethical considerations around our participants’ confidentiality, raw data cannot be shared publicly, but de-identified data may be available from the corresponding author upon reasonable request.

## Abbreviations

CFIR: Consolidated Framework for Implementation Research
EHE: Ending the HIV Epidemic
HCV: Hepatitis C virus
HIV: Human immunodeficiency virus
LGBTQ+: Lesbian, gay, bisexual, transgender, queer, etc.
PEP: Post-exposure prophylaxis
PrEP: Pre-exposure prophylaxis
PWUD: People who use drugs
SSP: Syringe services program
STIs: Sexually transmitted infections
